# Dependence of innate lymphoid cell 1 development on NKp46

**DOI:** 10.1371/journal.pbio.2004867

**Published:** 2018-04-27

**Authors:** Yufeng Wang, Wenjuan Dong, Yibo Zhang, Michael A. Caligiuri, Jianhua Yu

**Affiliations:** 1 The Ohio State University Comprehensive Cancer Center, Columbus, Ohio, United States of America; 2 The James Cancer Hospital, Columbus, Ohio, United States of America; 3 Division of Hematology, Department of Medicine, College of Medicine, The Ohio State University, Columbus, Ohio, United States of America; National Cancer Institute, United States of America

## Abstract

NKp46, a natural killer (NK) cell–activating receptor, is involved in NK cell cytotoxicity against virus-infected cells or tumor cells. However, the role of NKp46 in other NKp46^+^ non-NK innate lymphoid cell (ILC) populations has not yet been characterized. Here, an NKp46 deficiency model of natural cytotoxicity receptor 1 (*Ncr1*)^gfp/gfp^ and *Ncr1*^gfp/+^ mice, i.e., homozygous and heterozygous knockout (KO), was used to explore the role of NKp46 in regulating the development of the NKp46^+^ ILCs. Surprisingly, our studies demonstrated that homozygous NKp46 deficiency resulted in a nearly complete depletion of the ILC1 subset (ILC1) of group 1 ILCs, and heterozygote KO decreased the number of cells in the ILC1 subset. Moreover, transplantation studies confirmed that ILC1 development depends on NKp46 and that the dependency is cell intrinsic. Interestingly, however, the cell depletion specifically occurred in the ILC1 subset but not in the other ILCs, including ILC2s, ILC3s, and NK cells. Thus, our studies reveal that NKp46 selectively participates in the regulation of ILC1 development.

## Author summary

Group 1 innate lymphoid cells (ILCs) comprise two subsets: natural killer (NK) cells and ILC1s. Although NK cells and ILC1s are functionally distinct, a factor that regulates one subset but not the other has not been identified. In the current study, we discovered that NKp46, a marker expressed by both NK cells and ILC1s, is critical for the development of ILC1s but not NK cells. In mice lacking NKp46, and in wild-type (WT) mice depleted of immune cells by irradiation and then transplanted with bone marrow (BM) cells lacking NKp46, ILC1s that express cell surface receptor tumor necrosis factor (TNF)-related apoptosis-inducing ligand (TRAIL) were almost completely absent in all organs or tissues examined, including liver, spleen, BM, and small intestine (SI). In contrast, the cell number and signature cytokine expression of all other ILC subsets—namely NK cells, ILC2s, and ILC3s—were not significantly affected. Collectively, our findings provide new evidence supporting an essential role for NKp46 in the development of ILC1s.

## Introduction

Natural cytotoxicity receptor NKp46, encoded by the *Ncr1* gene, is a natural killer (NK) cell–activating receptor that plays roles in regulating the NK cell’s clearance of virus and rejection of tumor [1]. Following binding to its putative ligands, the receptor activates intracellular signaling through immune-receptor tyrosine-based activating motifs (ITAMs) [2]. Some non-NK innate lymphoid cell (ILC) populations also express NKp46, including the ILC1 subset (Lin^─^NKp46^+^NK1.1^+^CD49b^─^ CD49a^+^) [3] of group 1 ILCs and the ILC3 subset (Lin^─^CD127^+^RORγt^+^) of group 3 ILCs [4]. However, the role of NKp46 in these non-NK ILCs is still poorly understood. We previously reported that NKp46 defines a subset of NKT cells susceptible to malignant transformation in the presence of interleukin 15 (IL-15) and has a role in the NK cell clearance of herpes simplex virus 1 [5,6]. In the current study, we aimed to unravel the role of NKp46 in regulating the development and function of NKp46^+^ ILCs, especially ILC1s, using a genetic approach.

## Results and discussion

### ILC1s are absent in NKp46-deficient mice

**Fig 1:**
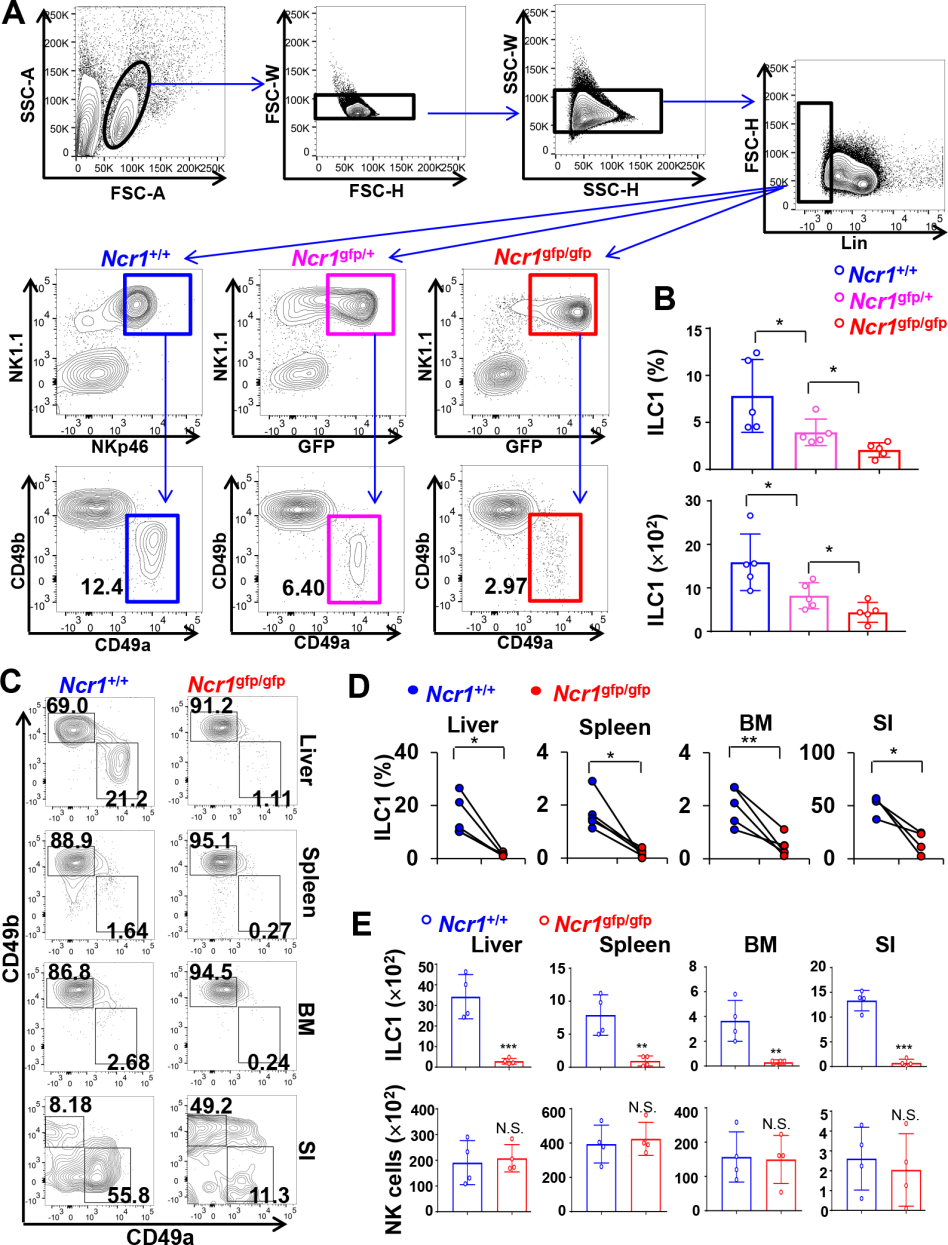
NKp46 is required for ILC1 development. (A) Gating strategy for ILC1s. ILC1s were gated on lymphocytes and then were further defined as Lin^−^NK1.1^+^NKp46^+^CD49b^─^CD49a^+^, with the exception that GFP^+^ was used to replace NKp46^+^ when *Ncr1* ^gfp/+^ or *Ncr1*^gfp/gfp^ mice were used. (B) Percentages or quantities of ILC1s were determined by flow cytometric analysis in the liver of *Ncr1*^gfp/gfp^, *Ncr1*^gfp/+^, and *Ncr1*^+/+^ mice. (C) NK cells were gated on Lin^─^NK1.1^+^NKp46^+^(or GFP^+^ for *Ncr1*^gfp/gfp^ mice)CD49b^+^CD49a^─^ among lymphocytes. ILC1s were gated on Lin^─^NK1.1^+^NKp46^+^(or GFP^+^ for *Ncr1*^gfp/gfp^ mice)CD49b^─^CD49a^+^ among lymphocytes. (D) Quantification of ILC1s in different organs or tissues in *Ncr1*^gfp/gfp^ mice and *Ncr1*^+/+^ littermates, i.e., summary data for (C). Each line demonstrates percentages of ILC1s for a pair of *Ncr1*^gfp/gfp^ and *Ncr1*^+/+^ littermates. (E) Quantities of NK cells or ILC1s in different organs of *Ncr1*^gfp/gfp^ mice and their *Ncr1*^+/+^ littermates (*n* = 4). Error bars, standard deviations; ***, *p* < 0.001; **, *p* < 0.01; *, *p* < 0.05. The numerical data for panels B, D and E can be found in S1 Data. Lin^─^, CD3^─^CD19^─^; BM, bone marrow; FSC-A, forward scatter area; FSC-H, FSC height; FSC-W, FSC width; GFP, green fluorescent protein; ILC1, innate lymphoid cell 1; NK, natural killer; *Ncr1*, natural cytotoxicity receptor 1; SI, small intestine; SSC-A, side scatter area; SSC-H, SSC height; SSC-W, SSC width.

An NKp46 knockout (KO) mouse model—in which *Ncr1*, the gene encoding NKp46, was replaced with green fluorescent protein (gfp) (*Ncr1*^gfp/gfp^) [5,7]—was used in this study. The development of ILC populations was assessed in different organs and tissues comparing wild-type (WT) (*Ncr1*^+/+^), heterozygous (*Ncr1*^gfp/+^), and KO (*Ncr1*^gfp/gfp^) mice. The gating strategy for ILC1 flow cytometric analysis is shown in Fig 1A. A clear NK1.1^+^NKp46^─^ population (more than 50%) was observed within the NK1.1^+^ population in the liver (S1A Fig), consistent with previous studies [8,9]. The intensity of CD49a surface expression was higher in the NK1.1^+^NKp46^+^CD49b^─^ CD49a^+^ ILC1 population than that in the NK1.1^+^NKp46^─^ CD49b^─^ CD49a^+^ population, and the latter population displayed a phenotype of CD3^─/dim^ (S1 and S2 Figs). Dadi and colleagues recently defined an ILC1-like population, named T cell receptor (TCR) lineage type 1 innate-like T cells (ILTC1), with a phenotype of NK1.1^+^NKp46^─^CD49a^+^ [10]. Moreover, NKp46 is considered a reliable marker for ILC1s and NK cells [9–11]. Thus, in the current study, the ILC1 subset was defined as NK1.1^+^NKp46^+^(or GFP^+^ for KO mice)CD49b^─^CD49a^+^ (Fig 1A), which does not include the NK1.1^+^NKp46^─^CD49b^─^CD49a^+^ population. It was surprising that, when compared to *Ncr1*^+/+^ littermates, proportionally the ILC1 subset (CD49b^─^CD49a^+^) was nearly completely absent in the liver of *Ncr1*^gfp/gfp^ mice and was significantly decreased in the liver of *Ncr1*^gfp/+^ mice (Fig 1A, bottom panel; Fig 1B, upper panel; S2 Fig, upper panel), while the percentage of the NK1.1^+^NKp46^─^(or GFP^─^ for KO mice)CD49b^─^CD49a^+^ population seems to be no different among the *Ncr1*^gfp/gfp^, *Ncr1*^+/gfp^, and *Ncr1*^+/+^ groups (S2 Fig, bottom panel). Furthermore, quantification of the ILC1 subset showed that the absolute cell quantity was also drastically reduced in the liver of the *Ncr1*^gfp/gfp^ mice and moderately reduced in the liver of *Ncr1*^gfp/+^ mice compared to their *Ncr1*^+/+^ littermates (Fig 1B, lower panel). We also used markers including CD62L, Eomesodermin (Eomes), and T-box expressed in T cells (T-bet) to confirm our observation regarding dependency on NKp46 for ILC1 development by comparing *Ncr1*^gfp/gfp^ mice and/or *Ncr1*^+/gfp^ mice to *Ncr1*^+/+^ mice (S3 and S4 Figs). The development of ILC populations was also assessed in other organs and tissues using the liver as our point of reference and comparing results in WT (*Ncr1*^+/+^) mice to *Ncr1*^gfp/gfp^ mice (Fig 1C and 1D). Cell quantification by flow cytometry indicated that ILC1s (CD49b^─^CD49a^+^) were nearly completely absent in the bone marrow (BM), spleen, and small intestine (SI) of *Ncr1*^gfp/gfp^ mice (Fig 1E). In contrast, the absolute number of NK cells, which are the main population that expresses NKp46 in tested organs or tissues, did not significantly change in *Ncr1*^gfp/gfp^ mice compared to their *Ncr1*^+/+^ littermates, consistent with a previous report [7] (Fig 1E, lower panel).

### Ncr1^gfp/gfp^ mice lack tumor necrosis factor (TNF)-related apoptosis-inducing ligand (TRAIL)^+^ ILC1s

**Fig 2:**
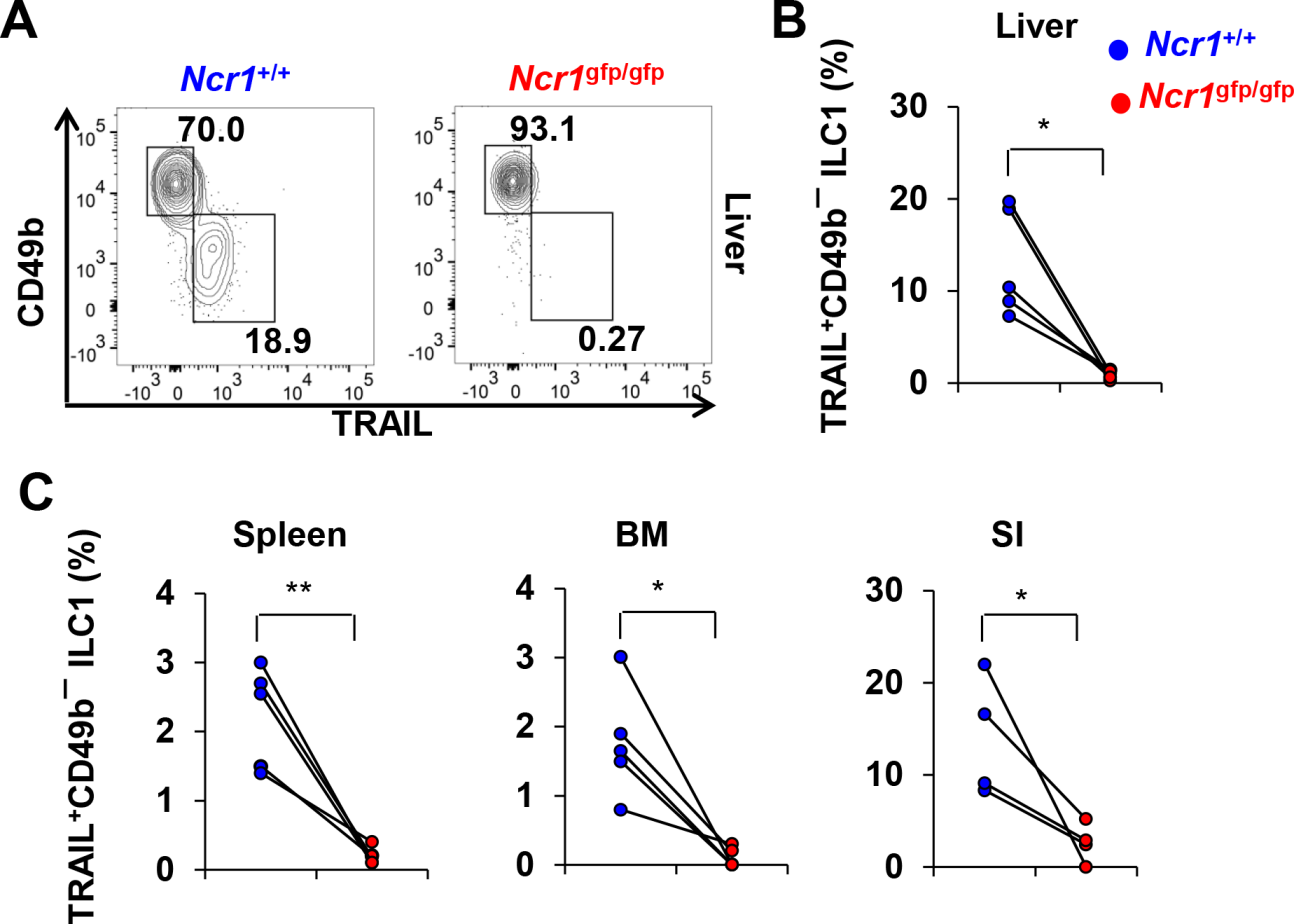
Absence of TRAIL^+^ ILC1s in NKp46-deficient mice. (A) Percentages of TRAIL^+^ ILC1s were analyzed by flow cytometric analysis in the liver of *Ncr1*^gfp/gfp^ mice and their *Ncr1*^+/+^ littermates. ILC1s were gated on Lin^─^NK1.1^+^NKp46^+^(or GFP^+^ for *Ncr1*^gfp/gfp^ mice) TRAIL^+^CD49b^─^ among lymphocytes. (B) Quantification of TRAIL^+^ ILC1s in the liver of *Ncr1*^gfp/gfp^ mice and their *Ncr1*^+/+^ littermates for (A) (*n* = 5). (C) Quantification of TRAIL^+^ ILC1s in other organs (spleen, *n* = 5; BM, *n* = 5; SI, *n* = 4) of *Ncr1*^gfp/gfp^ mice and their *Ncr1*^+/+^ littermates. **, *p* < 0.01; *, *p* < 0.05. The numerical data for panels B and C can be found in S1 Data. Lin^─^, CD3^─^CD19^─^; BM, bone marrow; GFP, green fluorescent protein; ILC1, innate lymphoid cell 1; *Ncr1*, natural cytotoxicity receptor 1; NK, natural killer; SI, small intestine; TRAIL, tumor necrosis factor–related apoptosis-inducing ligand.

TNF-related apoptosis-inducing ligand (TRAIL) is a functional protein, selectively expressed on the ILC1 subset, that plays an essential role in mediating the cytotoxicity of this population against target cells through triggering the death receptor-transduced signaling pathway [12]. The expression of TRAIL and lack of CD49b expression can also be used to distinguish ILC1s from NK cells [13] because resting NK cells in WT mice do not express TRAIL, while ILC1s do (Fig 2A, left). Consistent with the data shown in Fig 1 regarding the near-complete lack of CD49a^+^CD49b^─^ ILC1s, the TRAIL^+^CD49b^─^ population of NKp46^+^(or GFP^+^)NK1.1^+^ type I ILCs was barely detectable in the liver (Fig 2A and 2B) and other organs (Fig 2C) of *Ncr1*^gfp/gfp^ mice compared to their *Ncr1*^+/+^ littermate controls.

### NKp46 deficiency has no substantial adverse effect on ILC2 and ILC3 subsets

**Fig 3:**
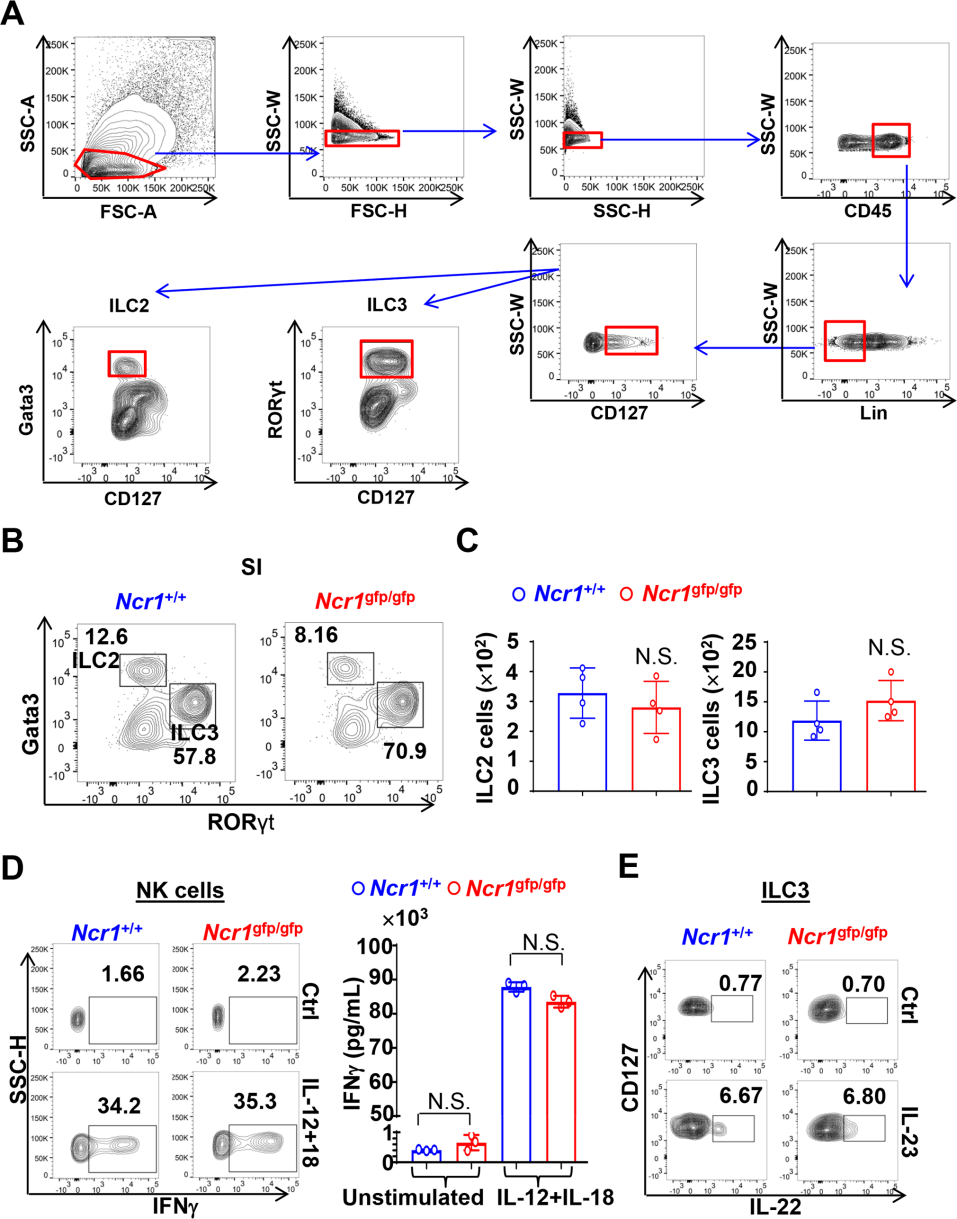
NKp46 deficiency does not affect ILC2s and ILC3s. (A) Gating strategy for ILC2s and ILC3s. ILC2s were gated on CD45^+^Lin^─^CD127^+^Gata3^+^, and ILC3s were gated on CD45^+^Lin^─^CD127^+^RORγt^+^. (B) Percentages of ILC2s or ILC3s were analyzed by flow cytometry in SI in *Ncr1*^gfp/gfp^ mice and *Ncr1*^+/+^ littermates. ILC2s were gated on CD45^+^Lin^─^CD127^+^Gata3^+^RORγt^─^ lymphocytes. ILC3s were gated on CD45^+^Lin^─^CD127^+^RORγt^+^Gata3^─^ lymphocytes. (C) Quantities of ILC2s or ILC3s were determined in SI of *Ncr1*^gfp/gfp^ mice and their *Ncr1*^+/+^ littermates (*n* = 4). (D) Lin^─^(or CD3^─^CD19^─^)NK1.1^+^NKp46^+^(or GFP^+^ for *Ncr1*^gfp/gfp^ mice)CD49b^+^ NK cells were sorted from the spleen of *Ncr1*^gfp/gfp^ mice or *Ncr1*^+/+^ littermates and were co-stimulated with IL-12 (10 ng/ml) and IL-18 (10 ng/ml) for 16 h, followed by the measurement of IFN-γ production by intracellular flow cytometric analysis (D, left panel, *n* = 3) or ELISA assays (D, right panel, *n* = 3). Golgi Plug was added at a 1:1,000 dilution to the culture 4 h prior to cell harvesting. (E) Homogenized SI cells isolated from *Ncr1*^gfp/gfp^ mice or *Ncr1*^+/+^ littermates were stimulated with IL-23 (10 ng/ml) for 4 h, followed by the measurement of IL-22 production by flow cytometric analysis after gating ILC3s on CD45^+^Lin^─^CD127^+^RORγt^+^. Golgi Plug was added at a 1:1,000 dilution to the culture 3 h prior to cell harvesting. Error bars, standard deviations. The numerical data for panel C and D can be found in S1 Data. ELISA, Enzyme-linked immunosorbent assay; FSC-A, forward scatter area; FSC-H, FSC height; IFN, interferon; IL, interleukin; ILC, innate lymphoid cell; *Ncr1*, natural cytotoxicity receptor 1; NK, natural killer; NS, no significance; RORγt, retinoic acid receptor (RAR) related orphan receptor gamma t; SI, small intestine; SSC-A, side scatter area; SSC-H, SSC height; SSC-W, SSC width.

Due to our observation that NKp46 deficiency restricted the development of ILC1s, we next set out to test whether this effect also occurred in other ILC subsets. However, using the gating strategy as described in Fig 3A, we did not observe a significant difference in the quantities or frequencies of Lin^─^CD127^+^Gata3^+^ ILC2s and Lin^─^CD127^+^RORγt^+^ ILC3s when *Ncr1*^gfp/gfp^ mice were compared to their *Ncr1*^+/+^ littermate controls (Fig 3B and 3C). There was an insufficient quantity of ILC1 cells from *Ncr1*^gfp/gfp^ mice to study how this NKp46 deficiency affects ILC1 function(s); however, we did observe that the interferon γ (IFN-γ) production by NK cells in response to co-stimulation with IL-12 and IL-18 was unaltered between cells isolated from *Ncr1*^gfp/gfp^ mice versus those from *Ncr1*^+/+^ littermate controls (Fig 3D). Likewise, IL-22 production by ILC3 cells isolated from *Ncr1*^gfp/gfp^ mice versus those from *Ncr1*^+/+^ littermate controls was not significantly different (Fig 3E). Consistent with our results, Satoh-Takayama and colleagues previously demonstrated that NKp46 is not required for IL-22-mediated intestinal innate immune cells in the gut to defend against *Citrobacter rodentium* [14]. Together, these results suggest that NKp46 does not control homeostasis or signature ILC cytokine production of ILC2s, NK cells, or ILC3s, but does selectively participate in the regulation of ILC1 development.

### NKp46 controls ILC1 development in a cell-intrinsic manner

**Fig 4:**
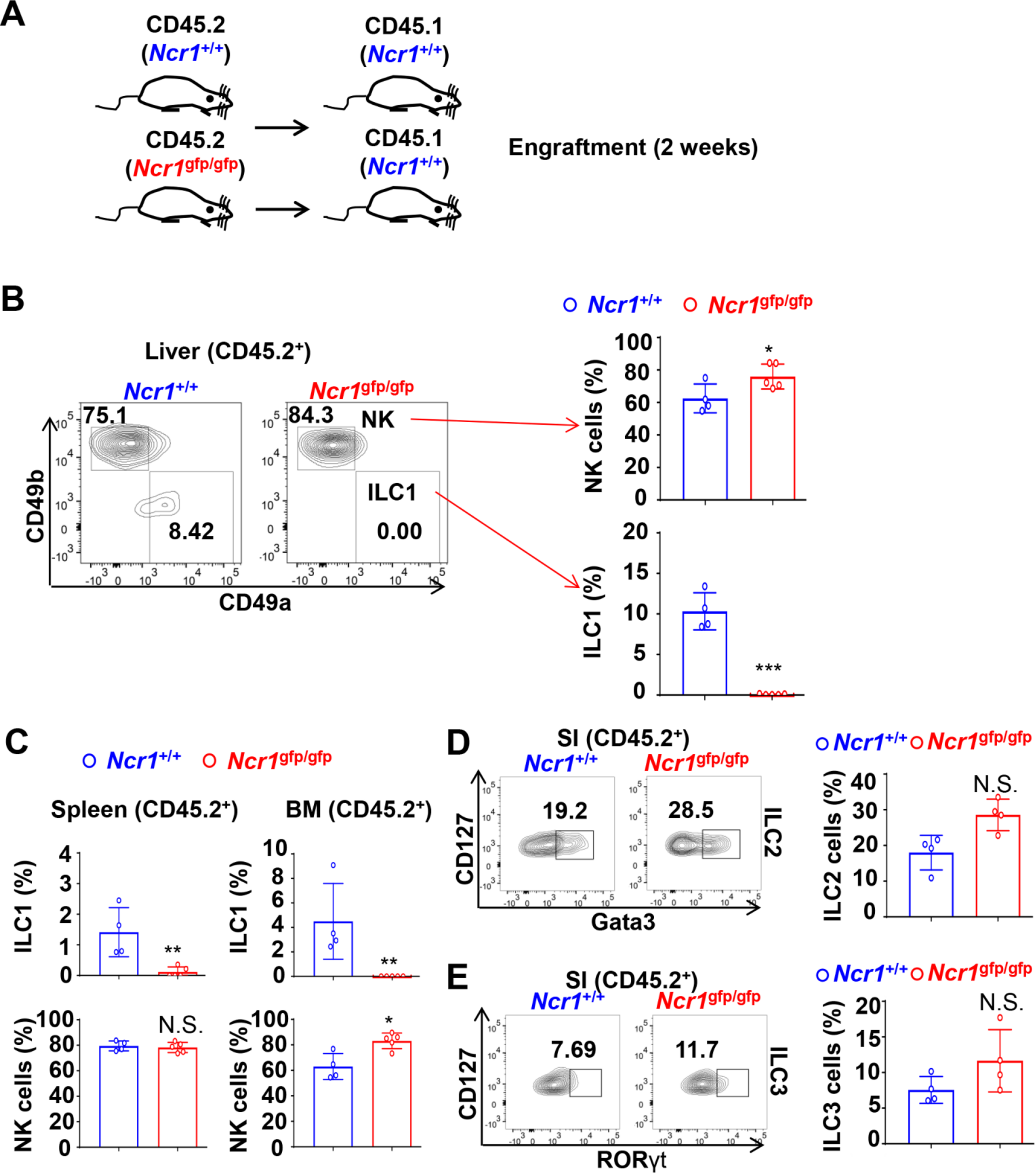
NKp46 plays a cell-intrinsic role in regulating ILC1 development. (A) Scheme of BM transplantation using BM cells of CD45.2 *Ncr1*^gfp/gfp^ mice or *Ncr1*^+/+^ littermate controls as donor cells to inject into CD45.1 recipients via tail vein. Development of ILC subsets was analyzed 2 weeks after transplantation. (B) Percentages of CD45.2^+^ NK cells or CD45.2^+^ ILC1s were analyzed by flow cytometric analysis in the liver of CD45.1 recipients, which were engrafted with BM cells of *Ncr1*^gfp/gfp^ mice (*n* = 5) or *Ncr1*^+/+^ littermates (*n* = 4). (C) Percentages of CD45.2^+^ NK cells or CD45.2^+^ ILC1s were analyzed in the spleen or BM of CD45.1 recipient mice, which were engrafted with BM cells of *Ncr1*^gfp/gfp^ mice (*n* = 5) or their *Ncr1*^+/+^ littermates (*n* = 4). (D and E) Data shown are representative dot plots of flow cytometric analysis (left panel) and summary data (right panel) of CD45.2^+^ILC2 (D) or CD45.2^+^ ILC3 (E) in SI in CD45.1 recipients, which were engrafted with BM cells of *Ncr1*^gfp/gfp^ mice (*n* = 4) or their *Ncr1*^+/+^ littermates (*n* = 4). Error bars, standard deviations; ***, *p* < 0.001; **, *p* < 0.01; *, *p* < 0.05. The numerical data for panels B, C, D and E can be found in S1 Data. Lin^─^, CD3^─^CD19^─^; BM, bone marrow; ILC, innate lymphoid cells; *Ncr1*, natural cytotoxicity receptor 1; NK, natural killer; RORγt, retinoic acid receptor (RAR) related orphan receptor gamma t; SI, small intestine.

Although NK cells and ILC1s are closely related, ILC1s do not develop through Lin^─^CD122^+^NK1.1^─^DX5^─^ NK cell precursors (NKPs) but can develop through Lin^─^c-kit^low^α4β7^+^CD127^+^CD25^─^ Flt3^─^ common helper innate lymphoid precursors (CHILPs). Both NKPs and CHILPs are derived from a common lymphoid progenitor (CLP) [15]. The proportion of both types of precursors are similar in the BM of *Ncr1*^gfp/gfp^ and *Ncr1*^+/+^ mice (S5 Fig). To validate that NKp46 deficiency results in the near-complete absence of ILC1s, and to test whether this phenomenon is cell intrinsic or extrinsic, BM transplantation was undertaken. For this purpose, irradiated WT CD45.1 recipients were engrafted with *Ncr1*^gfp/gfp^ (KO) or *Ncr1*^+/+^ (WT) CD45.2 donor BM cells (Fig 4A) or a 1:1 mixture of KO and WT (S6 Fig) via a tail-vein injection. Two weeks later, quantification of various ILC subsets was undertaken by flow cytometric analysis. Donor cells and host cells were distinguished by staining cells with an anti-CD45.2 antibody. We found that there was a nearly complete absence of ILC1s in the liver, spleen, and BM of WT recipients when *Ncr1*^gfp/gfp^ BM cells were used as donor cells. However, the ILC1 population was present in significantly larger quantities when *Ncr1*^+/+^ BM cells were used as donor cells (Fig 4B and 4C and S6 Fig). In contrast, the quantity of ILC2 cells and ILC3 cells in the SI was not affected in recipient mice engrafted with *Ncr1*^gfp/gfp^ or *Ncr1*^+/+^ BM donor cells (Fig 4D and 4E). The moderate increase in the proportion of NK cells could be occurring due to the complete lack of ILC1s among NKp46^+^NK1.1^+^ type I ILCs (Fig 4B top right; Fig 4C bottom right). Collectively, these results further confirm that ILC1 development depends on NKp46, and this dependency is cell-intrinsic (Fig 1).

In conclusion, our findings provide novel evidence that NKp46 plays a critical role in ILC1 development. Previous studies in this area focused on transcriptional control of ILC development. It is known that T-bet and Eomes regulate NK cell development, Gata3 controls ILC2 development, and RORγt defines the ILC3 lineage [15]. Several transcription factors—such as nuclear factor interleukin 3 regulated (Nfil3), runt related transcription factor 3 (Runx3), and T-bet—have been found to control ILC1 development; however, these factors do not play a selective role in determining ILC1 development [15]. That is, these transcription factors have some overlapping roles in several types of ILCs and thus individually cannot determine the fate of ILC1 development. For example, T-bet has been shown to play a role not only in ILC1 development but also in NK cell and ILC3 development [15]. Here, we identified a receptor that selectively determines the developmental fate of ILC1s. Our study also supports the notion that ILC1s and NK cells belong to different lineages, although both belong to group 1 ILCs, and both can produce IFN-γ when activated (e.g., by cytokines). Our study also shows that *Ncr1*^gfp/gfp^ mice may serve as a useful animal model for investigating the physiological or pathological functions of ILC1s, given their near-complete absence in various organs, while the development and function of other ILCs are kept nearly intact, with the exception of functions related to NKp46’s role in NK cells [7,16].

## Materials and methods

### Ethics statement

All animal experiments were performed according to the protocol (# 2012A00000090), which has been approved by The Ohio State University Institutional Animal Care and Use Committee (IACUC). No human subjects were involved in this study.

### Mice

NKp46 KO C57BL/6 mice with *Ncr1* replaced with gfp (*Ncr1*^gfp/gfp^) [5,7] and their heterozygous littermates (*Ncr1*^gfp/+^) were used. NKp46 KO C57BL/6 mice were previously described [7]. Congenic CD45.1^+^ C57BL/6 mice for transplant experiments were purchased from Jackson Laboratory. All mice used in these studies were 10–12 weeks of age.

### Flow cytometric analysis and cell sorting

Cells were labelled with flow antibodies for 15 minutes in the dark in PBS containing 2% BSA. Labelled cells were washed twice and resuspended in PBS containing 2% BSA. The prepared samples were analyzed using an LSR-II flow cytometer (BD Bioscience) or sorted using an Aria II cell sorter (BD Bioscience). Anti-CD19-PeCy7 (561739), anti-CD3-PeCy7 (552774), anti-NK1.1-BV421 (562921), anti-NKp46-FITC (560756), anti-NKp46-AF647 (560755), anti-CD49b-PE (553858), anti-CD49a-PerCP-Cy5.5 (564862), anti-CD62L-APC (553152), anti-T-bet-APC (561264), anti-CD45.2-AF700 (560693), anti-CD45.2-FITC (553772), anti-Gata3-AF647 (560068), anti-RORγt-PE (562607), anti-CD127-V450 (561205), anti-CD117-PE (553869), anti-LPAM-1-APC (562376), anti-Flt3-BV421 (566292), anti-Ly-6A/E-APC (565355), and anti-CD122-PE (553362) were purchased from BD Bioscience. Anti-IFN-γ-AF-700 (505823) and anti-CD25-Pacific Blue (102022) were purchased from Biolegend. Anti-CD253-APC (17-5951-82), anti-Eomes-PE (12-4875-82), and anti-CD127-PerCP-Cy5.5 (45-1271-80) were purchased from eBioscience.

### BM transplantation

For BM cell transplantation, CD45.2^+^ donor BM cells collected from *Ncr1*^gfp/gfp^ mice (5×10^6^), *Ncr1*^+/+^ mice (5×10^6^), or a mixture of these 2 types of cells at a ratio of 1:1 (10×10^6^ in total) were injected IV into the CD45.1^+^ congenic recipient mice, which were lethally irradiated (4 cGy twice on the same day) using an X-ray irradiator. The detailed protocol was described in our previous studies [17,18].

### Enzyme-linked immunosorbent assay (ELISA)

1 × 10^5^ NK cells were seeded into a 96-well plate and cultured with or without IL-12 plus IL-18 for 24 h. Supernatants were then harvested to detect IFN-γ production, which was assessed by the Mouse IFN-gamma Uncoated ELISA Kit (Catalog #88-7314-88, Invitrogen)

### Statistics

Student's *t* test or paired *t* test was used to analyze two independent or paired groups, respectively. A *p* value less than 0.05 was considered statistically significant.

## Supporting information

S1 Fig Characterization of the Lin^─^NK1.1^+^NKp46^─^CD49a^+^ subset in WT mice. (A) NKp46^+^ and NKp46^─^ subsets within the NK1.1^+^ population. Compared to the NK1.1^+^NKp46^+^CD49a^+^ ILC1 subset, the NK1.1^+^NKp46^─^CD49a^+^ subset in the liver has lower expression of the surface protein CD49a. (B) The NK1.1^+^NKp46^─^ subset displayed a CD3^-/dim^ phenotype. The numbers in the quadrants of flow figures are percentages for different cell populations. FSC-A, forward scatter area; FSC-H, FSC height; FSC-W, FSC width; MFI, median fluorescence intensity; NK, natural killer; SSC-A, side scatter area; SSC-H, SSC height; SSC-W, SSC width; WT, wild type.
(PPTX)

S2 Fig Comparison of the Lin^─^NK1.1^+^NKp46^─^CD49b^─^CD49a^+^ subset and the Lin^─^NK1.1^+^NKp46^+^CD49b^─^CD49a^+^ (ILC1) subset in the liver among *Ncr1*^+/+^ (WT), *Ncr1*^+/gfp^ [heterozygous KO], and *Ncr1*^gfp/gfp^ (homozygous KO) mice. Gating strategy is shown in Fig 1A. While the ILC1 subset was decreased in *Ncr1*^+/gfp^ and *Ncr1*^gfp/gfp^ mice compared to *Ncr1*^+/+^ mice, relative proportion of NK1.1^+^NKp46^─^(or GFP^─^ for KO mice)CD49b^─^CD49a^+^ population was not changed among the *Ncr1*^+/+^, *Ncr1*^+/gfp^, and *Ncr1*^gfp/gfp^ mice. The numbers in the quadrants of flow figures are percentages of indicated cell populations. GFP, green fluorescent protein; ILC1, innate lymphoid cell 1; KO, knockout; *Ncr1*, natural cytotoxicity receptor 1; NK, natural killer; WT, wild type.
(PPTX)

S3 Fig CD62L^─^ ILC1s were significantly reduced in the liver of NKp46-deficient mice. (A) CD62L^─^ cells were gated on Lin^─^NK1.1^+^NKp46^+^(GFP^+^ for KO mice). Gating strategy was shown in Fig 1A. (B) A summary analysis was performed for data in (A) (*n* = 3). The numbers in the quadrants of flow figures are percentages of indicated cell populations. Error bars, standard deviations; **, *p* < 0.01. The numerical data for panel B can be found in S1 Data. GFP, green fluorescent protein; ILC1, innate lymphoid cell 1; KO, knockout; *Ncr1*, natural cytotoxicity receptor 1; NK, natural killer.
(PPTX)

S4 Fig T-bet^+^Eomes^─^ ILC1s were significantly reduced in the liver of NKp46-deficient mice. (A) T-bet^+^Eomes^─^ cells were gated on Lin^─^NK1.1^+^NKp46^+^(GFP^+^ for KO mice). (B) A summary analysis was performed for (A) (*n* = 3). The numbers in the quadrants of flow figures are percentages for different cell populations. Error bars, standard deviations; **, *p* < 0.01; **, *p* < 0.01. *Ncr1*^gfp/gfp^ mice were excluded in this experiment because intracellular staining to determine the expression of transcription factors T-bet and Eomes resulted in quenching of GFP fluorescence. The numerical data for panel B can be found in S1 Data. Eomes; Eomesodermin; FSC-H, forward scatter height; FSC-W, FSC width; GFP, green fluorescent protein; ILC1, innate lymphoid cell 1; KO, knockout; *Ncr1*, natural cytotoxicity receptor 1; NK, natural killer; SSC-A, scatter side area; SSC-H, SSC height; T-bet, T-box expressed in T cells.
(PPTX)

S5 Fig Analysis of ILC precursors in BM of *Ncr1*^gfp/gfp^ mice or *Ncr1*^+/+^ littermates. (A, B) Lin^−^C-kit^low^α4β7^+^CD127^+^CD25^−^Flt3^−^ CHILPs (A) and Lin^−^CD122^+^NK1.1^−^DX5^−^NKPs (B) in the bone marrow from *Ncr1*^gfp/gfp^ mice or *Ncr1*^+/+^ littermates were detected by flow cytometric analysis. BM, bone marrow; CHILPs, common helper innate lymphoid precursors; GFP, green fluorescent protein; *Ncr1*; natural cytotoxicity receptor 1; NK, natural killer; NKPs, NK cell precursors.
(PPTX)

S6 Fig The cell-intrinsic role of NKp46 in regulating ILC1 development. (A) Scheme of BM transplantation using BM cells from CD45.2 *Ncr1*^gfp/gfp^ mice and *Ncr1*^+/+^ littermate controls as donor cells. A mixture of BM cells from CD45.2 *Ncr1*^gfp/gfp^ mice and *Ncr1*^+/+^ littermate controls was used at a 1:1 ratio and injected into CD45.1 recipients via tail vein. The development of the ILC subset in the liver was analyzed 2 weeks after transplantation. (B) ILC1s were initially gated on Lin^−^NK1.1^+^NKp46^+^(GFP^+^ for KO mice) and were further defined by CD49a and CD49b surface expression. (C) Percentages of CD45.2^+^ ILC1s were analyzed by flow cytometry in the liver of CD45.1 recipients, which were engrafted with BM cells from *Ncr1*^gfp/gfp^ mice or *Ncr1*^+/+^ littermates (*n* = 5). ***, *p* < 0.001. The numerical data for panel C can be found in S1 Data. BM, bone marrow; FSC-A; forward scatter area; FSC-H; FSC height; FSC-W; FSC width; GFP, green fluorescent protein; ILC, innate lymphoid cell; KO, knockout; *Ncr1*, natural cytotoxicity receptor 1; NK, natural killer; SSC-A, side scatter area; SSC-H, SSC height; SSC-W, SSC width.
(PPTX)

S1 Data Numerical values underlying the graphs in Figs 1, 2, 3 and 4 and in S3, S4 and S6 Figs. BM, bone marrow; Eomes, Eomesodermin; GFP, green fluorescent protein; IL, interleukin; ILC, innate lymphoid cell; *Ncr1*, natural cytotoxicity receptor 1; NK, natural killer; SI, small intestine; T-bet, T-box expressed in T cells.
(XLSX)

## Abbreviations

BM: bone marrow
CHILP: common helper innate lymphoid precursor
CLP: common lymphoid progenitor
ELISA: enzyme-linked immunosorbent assay
Eomes: Eomesodermin
GFP: green fluorescent protein
IFN: interferon
IL: interleukin
ILC: innate lymphoid cell
ITAM: immune-receptor tyrosine-based activating motifs
KO: knockout
Ncr1: natural cytotoxicity receptor 1
Nfil3: nuclear factor interleukin 3 regulated
NK: natural killer
NKP: NK cell precursor
RORγt: retinoic acid receptor (RAR) related orphan receptor gamma t
Runx3: runt-related transcription factor 3
SI: small intestine
T-bet: T-box expressed in T cells
TCR: T cell receptor
TRAIL: tumor necrosis factor–related apoptosis-inducing ligand
WT: wild type
