# Supplementary figures and images for: Dependence of innate lymphoid cell 1 development on NKp46
Source: PLoS Biol. 2018 Apr 27;16(4):e2004867. doi: 10.1371/journal.pbio.2004867 (PMC5922978; doi:10.1371/journal.pbio.2004867)

## Slide 1
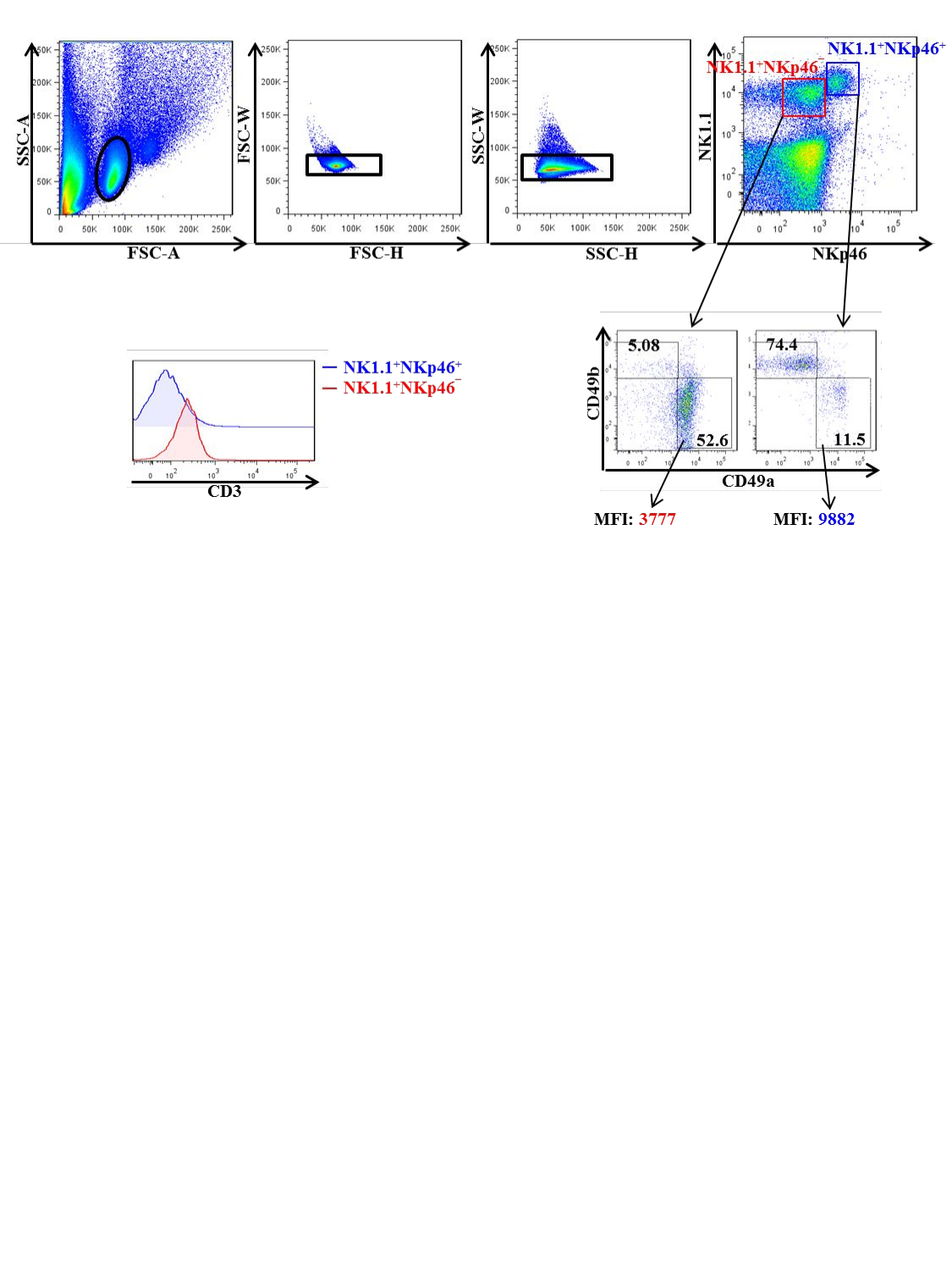

Supplement: S1 Fig — (A) NKp46+ and NKp46─ subsets within the NK1.1+ population. Compared to the NK1.1+NKp46+CD49a+ ILC1 subset, the NK1.1+NKp46─CD49a+ subset in the liver has lower expression of the surface protein CD49a. (B) The NK1.1+NKp46─ subset displayed a CD3-/dim phenotype. The numbers in the quadrants of flow figures are percentages for different cell populations. FSC-A, forward scatter area; FSC-H, FSC height; FSC-W, FSC width; MFI, median fluorescence intensity; NK, natural killer; SSC-A, side scatter area; SSC-H, SSC height; SSC-W, SSC width; WT, wild type. (PPTX) [file pbio.2004867.s001.pptx]

## Slide 1
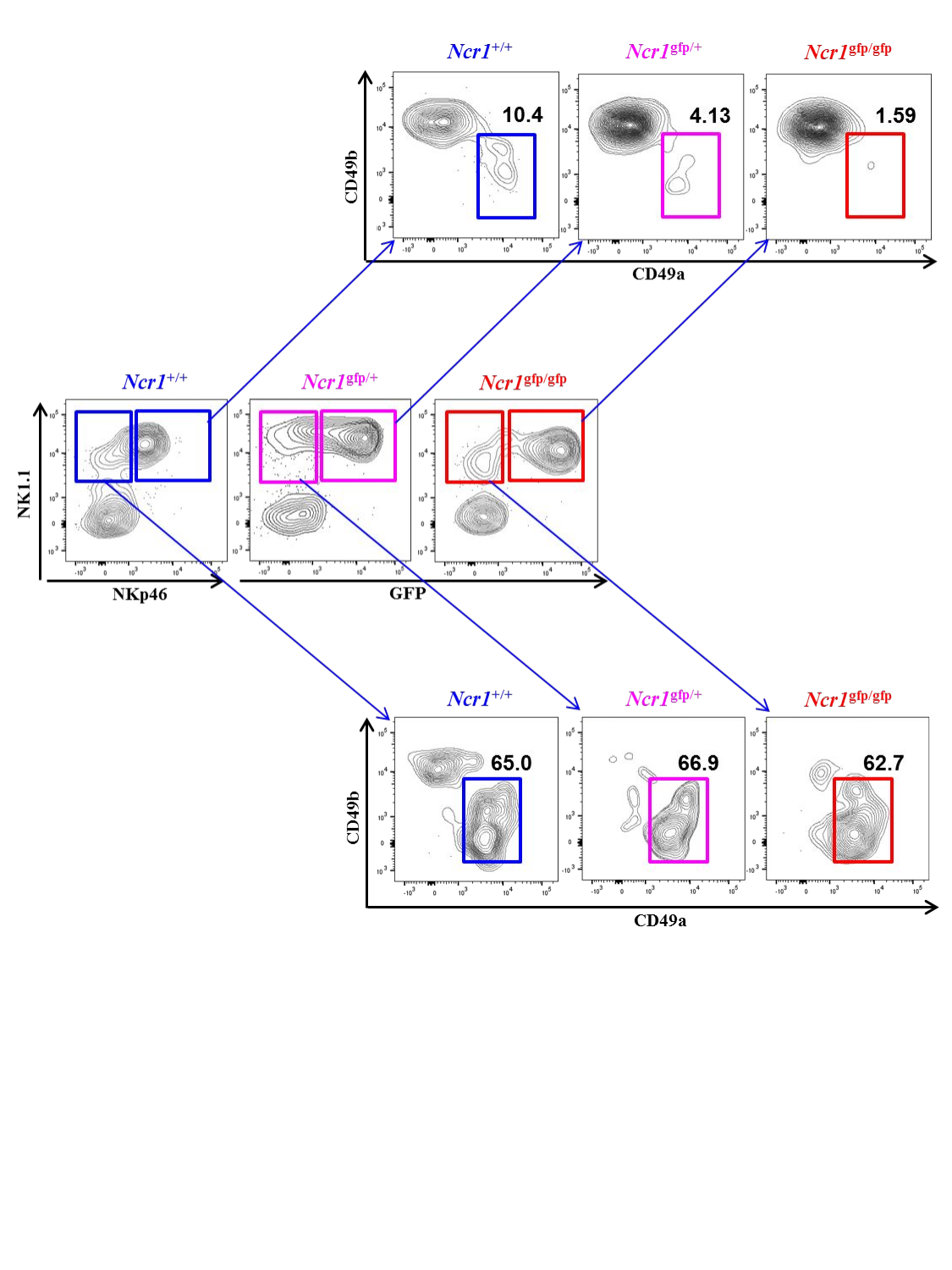

Supplement: S2 Fig — Gating strategy is shown in Fig 1A. While the ILC1 subset was decreased in Ncr1+/gfp and Ncr1gfp/gfp mice compared to Ncr1+/+ mice, relative proportion of NK1.1+NKp46─(or GFP─ for KO mice)CD49b─CD49a+ population was not changed among the Ncr1+/+, Ncr1+/gfp, and Ncr1gfp/gfp mice. The numbers in the quadrants of flow figures are percentages of indicated cell populations. GFP, green fluorescent protein; ILC1, innate lymphoid cell 1; KO, knockout; Ncr1, natural cytotoxicity receptor 1; NK, natural killer; WT, wild type. (PPTX) [file pbio.2004867.s002.pptx]

## Slide 1
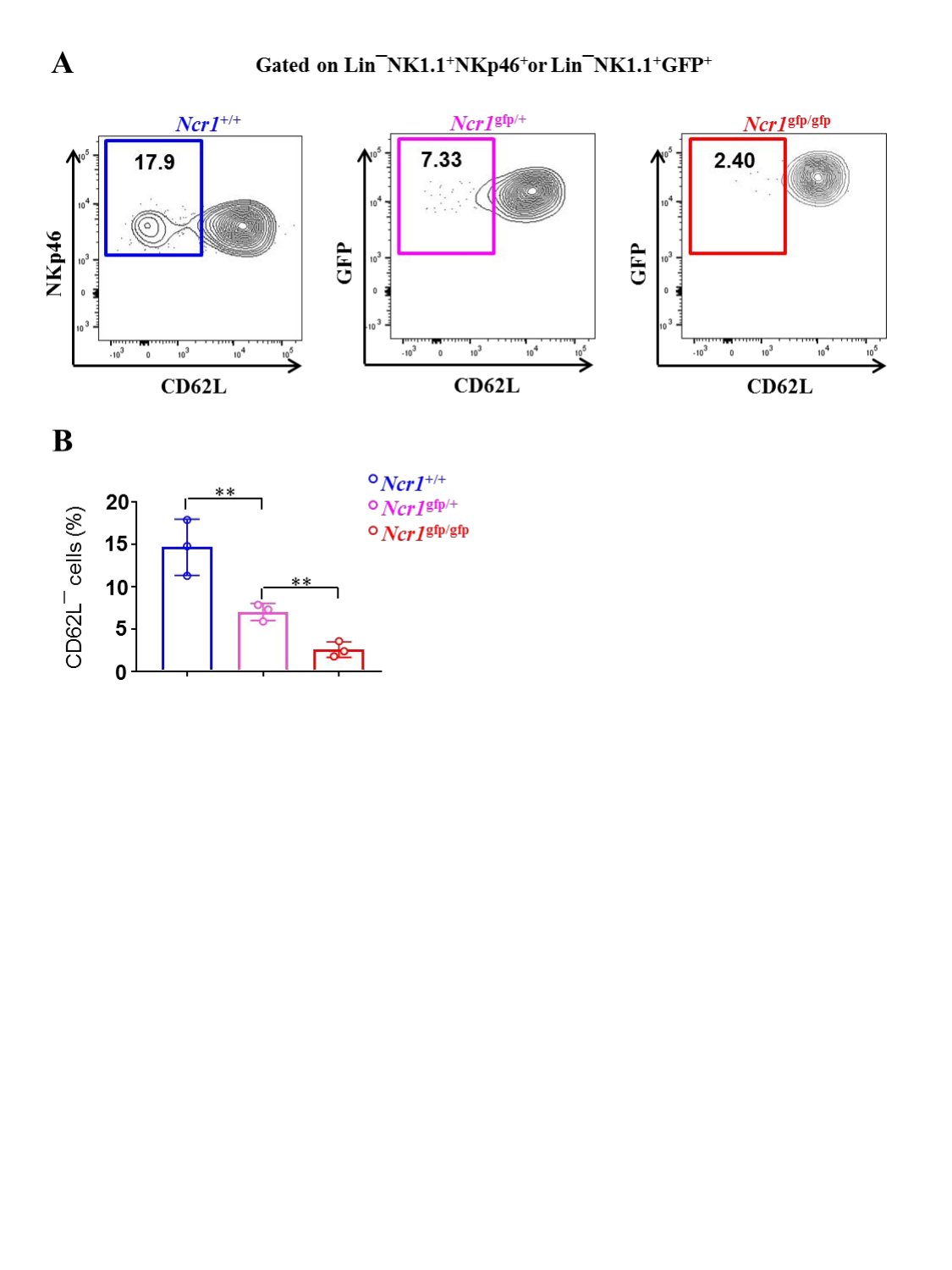

Supplement: S3 Fig — (A) CD62L─ cells were gated on Lin─NK1.1+NKp46+(GFP+ for KO mice). Gating strategy was shown in Fig 1A. (B) A summary analysis was performed for data in (A) (n = 3). The numbers in the quadrants of flow figures are percentages of indicated cell populations. Error bars, standard deviations; **, p < 0.01. The numerical data for panel B can be found in S1 Data. GFP, green fluorescent protein; ILC1, innate lymphoid cell 1; KO, knockout; Ncr1, natural cytotoxicity receptor 1; NK, natural killer. (PPTX) [file pbio.2004867.s003.pptx]

## Slide 1
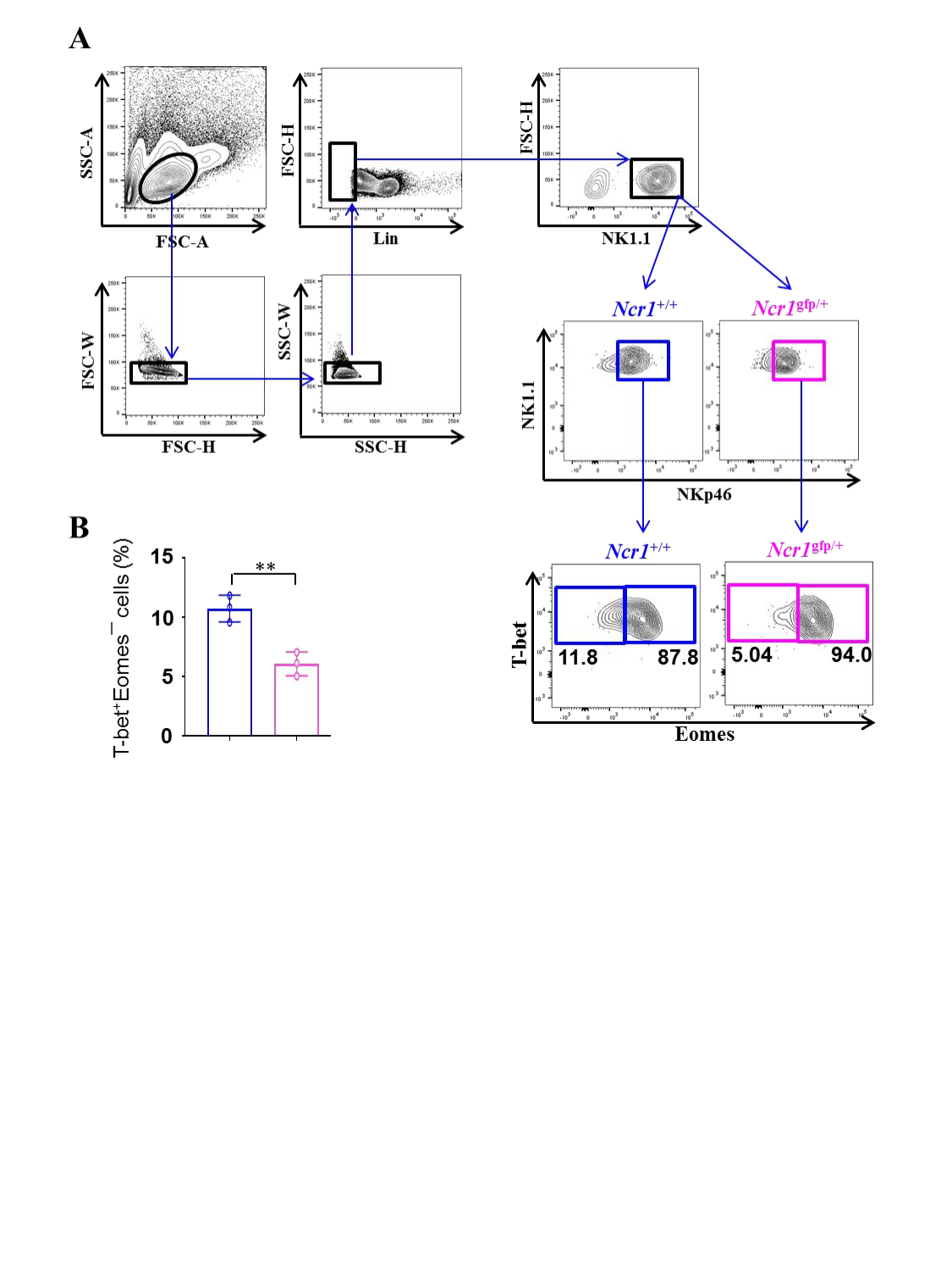

Supplement: S4 Fig — (A) T-bet+Eomes─ cells were gated on Lin─NK1.1+NKp46+(GFP+ for KO mice). (B) A summary analysis was performed for (A) (n = 3). The numbers in the quadrants of flow figures are percentages for different cell populations. Error bars, standard deviations; **, p < 0.01; **, p < 0.01. Ncr1gfp/gfp mice were excluded in this experiment because intracellular staining to determine the expression of transcription factors T-bet and Eomes resulted in quenching of GFP fluorescence. The numerical data for panel B can be found in S1 Data. Eomes; Eomesodermin; FSC-H, forward scatter height; FSC-W, FSC width; GFP, green fluorescent protein; ILC1, innate lymphoid cell 1; KO, knockout; Ncr1, natural cytotoxicity receptor 1; NK, natural killer; SSC-A, scatter side area; SSC-H, SSC height; T-bet, T-box expressed in T cells. (PPTX) [file pbio.2004867.s004.pptx]

## Slide 1
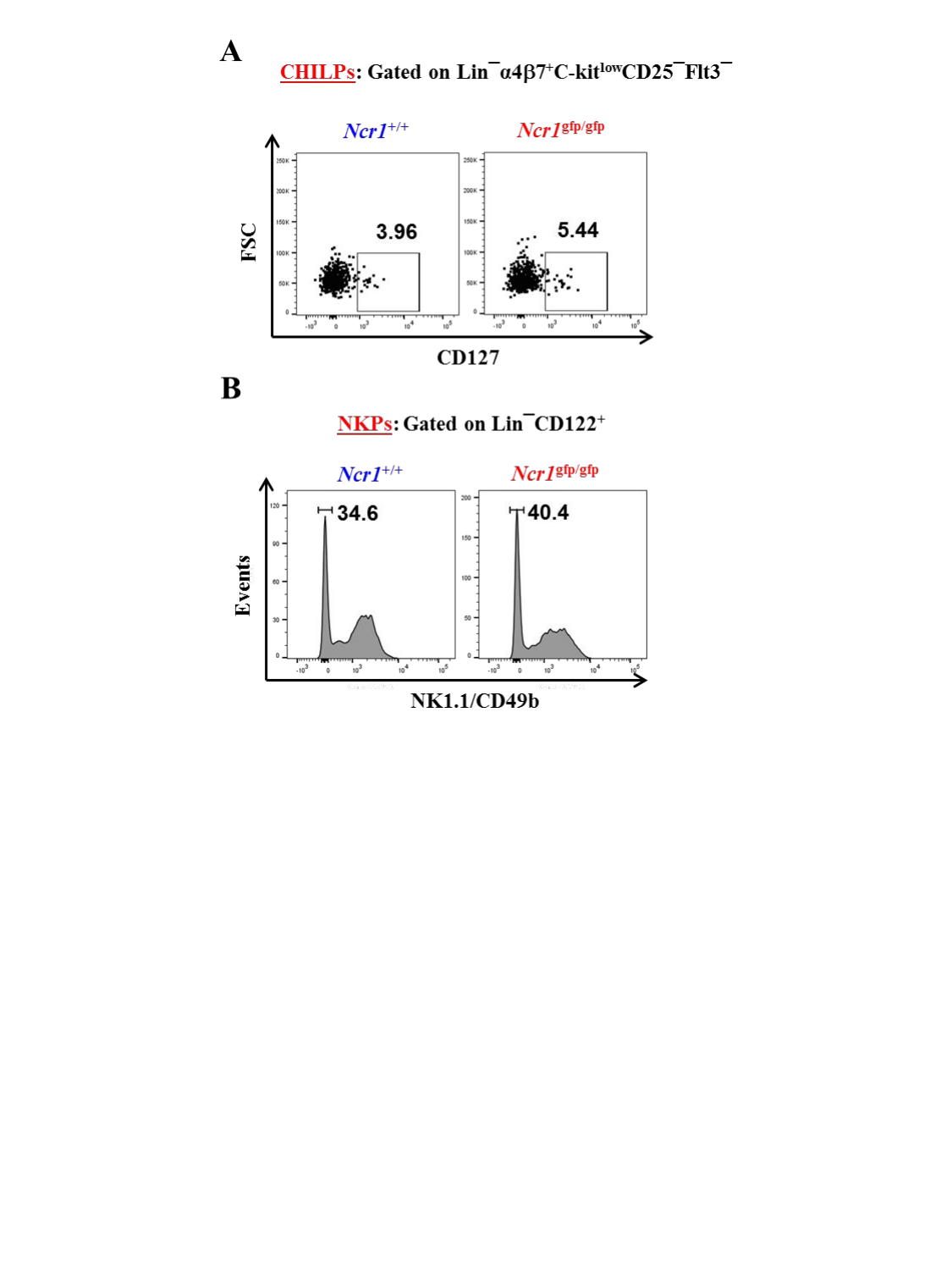

Supplement: S5 Fig — (A, B) Lin−C-kitlowα4β7+CD127+CD25−Flt3− CHILPs (A) and Lin−CD122+NK1.1−DX5−NKPs (B) in the bone marrow from Ncr1gfp/gfp mice or Ncr1+/+ littermates were detected by flow cytometric analysis. BM, bone marrow; CHILPs, common helper innate lymphoid precursors; GFP, green fluorescent protein; Ncr1; natural cytotoxicity receptor 1; NK, natural killer; NKPs, NK cell precursors. (PPTX) [file pbio.2004867.s005.pptx]

## Slide 1
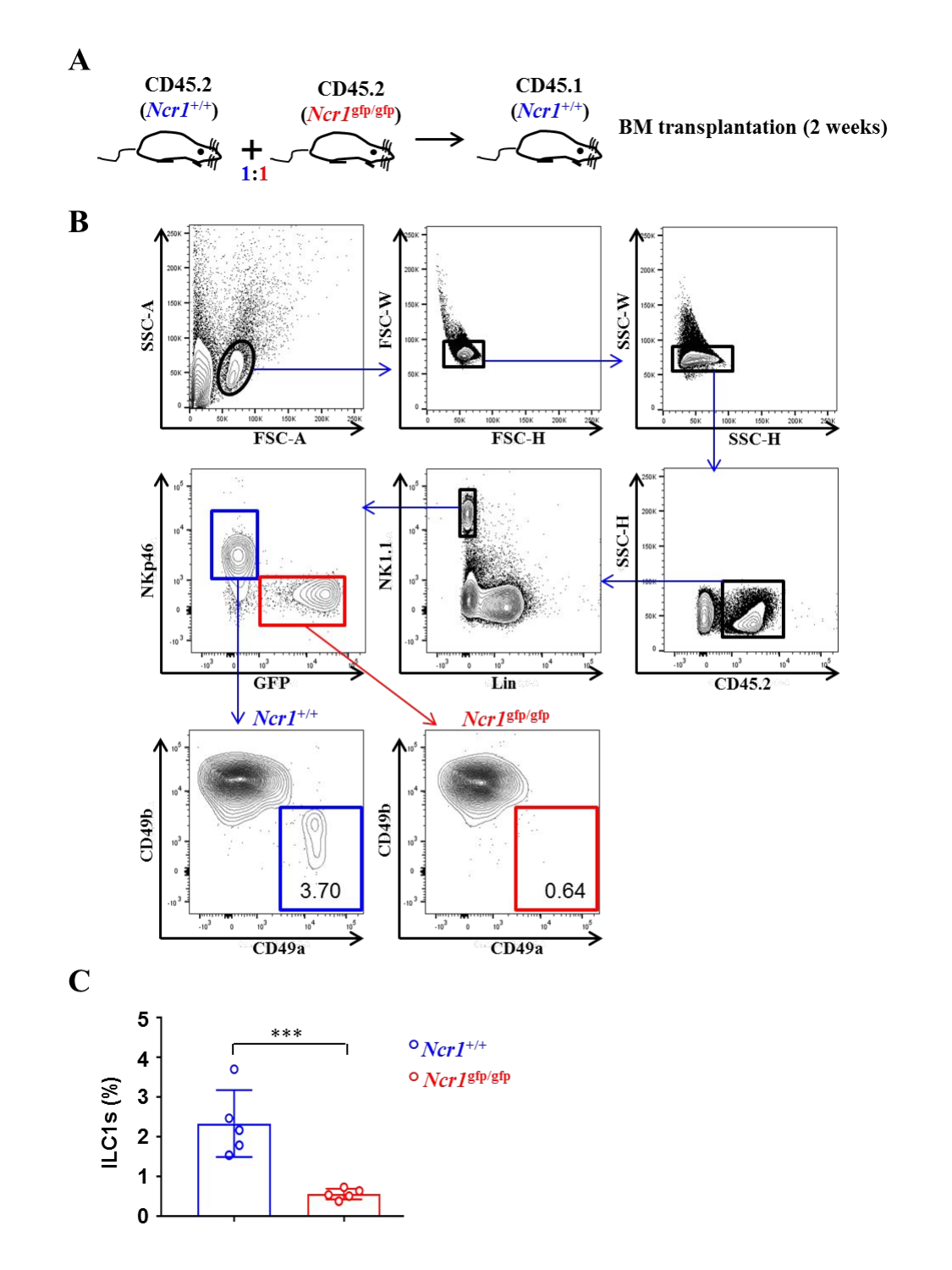

Supplement: S6 Fig — (A) Scheme of BM transplantation using BM cells from CD45.2 Ncr1gfp/gfp mice and Ncr1+/+ littermate controls as donor cells. A mixture of BM cells from CD45.2 Ncr1gfp/gfp mice and Ncr1+/+ littermate controls was used at a 1:1 ratio and injected into CD45.1 recipients via tail vein. The development of the ILC subset in the liver was analyzed 2 weeks after transplantation. (B) ILC1s were initially gated on Lin−NK1.1+NKp46+(GFP+ for KO mice) and were further defined by CD49a and CD49b surface expression. (C) Percentages of CD45.2+ ILC1s were analyzed by flow cytometry in the liver of CD45.1 recipients, which were engrafted with BM cells from Ncr1gfp/gfp mice or Ncr1+/+ littermates (n = 5). ***, p < 0.001. The numerical data for panel C can be found in S1 Data. BM, bone marrow; FSC-A; forward scatter area; FSC-H; FSC height; FSC-W; FSC width; GFP, green fluorescent protein; ILC, innate lymphoid cell; KO, knockout; Ncr1, natural cytotoxicity receptor 1; NK, natural killer; SSC-A, side scatter area; SSC-H, SSC height; SSC-W, SSC width. (PPTX) [file pbio.2004867.s006.pptx]
